# Sex and Gender Aspects for Patient Stratification in Allergy Prevention and Treatment

**DOI:** 10.3390/ijms21041535

**Published:** 2020-02-24

**Authors:** Massimo De Martinis, Maria Maddalena Sirufo, Mariano Suppa, Daniela Di Silvestre, Lia Ginaldi

**Affiliations:** 1 Department of Life, Health and Environmental Sciences, University of L’Aquila, 67100 L’Aquila, Italy; maddalena.sirufo@gmail.com (M.M.S.); daniela.disilvestre@aslteramo.it (D.D.S.); lia.ginaldi@cc.univaq.it (L.G.); 2 Allergy and Clinical Immunology Unit, Center for the diagnosis and treatment of Osteoporosis, AUSL 04 Teramo, Italy; 3 Department of Dermatology, Hôpital Erasme, Université Libre de Bruxelles, 1070 Brussels, Belgium; dr.marianosuppa@gmail.com

**Keywords:** allergy, gender, sex, sex hormones, IgE, hypersensitivity, immunity, asthma, atopic dermatitis, rhinitis, urticarial, prevention, treatment, patient stratification

## Abstract

Allergies are rapidly worsening in recent decades, representing the most common immunological diseases. The mechanism of disorders such as asthma, rhinocongiuntivitis, urticaria, atopic dermatitis, food and drug allergies, and anaphylaxis still remain unclear and consequently treatments is mostly still symptomatic and aspecific while developments of new therapies are limited. A growing amount of data in the literature shows us how the prevalence of allergic diseases is different in both sexes and its changes over the course of life. Genes, hormones, environmental and immunological factors affect sex disparities associated with the development and control of allergic diseases, while they more rarely are considered and reported regarding their differences related to social, psychological, cultural, economic, and employment aspects. This review describes the available knowledge on the role of sex and gender in allergies in an attempt to improve the indispensable gender perspective whose potential is still underestimated while it represents a significant turning point in research and the clinic. It will offer insights to stimulate exploration of the many aspects still unknown in this relationship that could ameliorate the preventive, diagnostic, and therapeutic strategies in allergic diseases.

## 1. Introduction

The differences in biological sex, gender identity, relations, role, and their impact on health and diseases may have significative implications for prevention, screening, diagnosis and treatment. Based on this assumption gender medicine represent an innovative approach to the practice of clinical medicine and medical research.

Currently, the concepts of gender medicine, the personalization of care, and of precision medicine are closely linked, with gender medicine having become synonymous with better medicine for all. A new conception of medicine based on the promotion of health and on the appropriateness of care, in fact, has been accompanied, in the last decade, by growing attention to sex and gender differences. Use of the sex-gender perspective in clinical practice and medical research, as well as in the planning and management of health programs in particular, has progressively been recognized as an element of innovation. Nowadays there is consolidated scientific evidence that men and women not only present different clinical and symptomatic manifestations for the same pathology, but develop substantially different therapeutic responses. Gender medicine focuses not only on sex differences (defined by reproductive organs, sex hormones levels, and differential organization of chromosomes) and on their impact in the development, diagnosis, and treatment of diseases, but also considers gender differences as social and cultural determinants that can have a decisive impact on health. Gender-specific differences pay attention to people living circumstances related to cultural, social, economic, and working conditions. Sex and gender are multi-dimensional, entangled, interactive, and sometimes are difficult to separate, so the use of both words sex-gender may be helpful in order to grasp the meaning of both the social and biologic context [1,2,3].

Sex-gender aspects should become essential stratification parameters in allergology for targeted diagnostics and tailored treatments together with molecular, genetic, and epigenetic aspects. Allergic diseases such as food allergies, atopic dermatitis, and allergic asthma with almost one billion cases worldwide, have an evident differential prevalence among women and men. They, in general, initially affect young males more than females, more than in post pubertal females where the incidence of allergies increases to become superior or equivalent to that observed in post pubertal males. IgE sensitization affects males and females differently: the former show a significantly higher prevalence. Until puberty, they also show a higher prevalence of clinical manifestations, but after puberty females overtake them in the incidence of allergic symptoms. The IgE levels are influenced by the mestrual cycle, suggesting a role of sexual hormones [4]. Female and male differences in allergic responses may be affected by both gender and sex. Exposure, recognition, and clearance of allergens are influenced by physiological and anatomical sexual differences, while gender may reflect behaviors that influence exposure to allergens, access to healthcare, or health-seeking behaviors that affect the course of the reaction. Gender-specific differences pay attention to people living circumstances related to cultural, social, economic, and working conditions. Although many years have passed since the first sex-gender approaches in medicine and the medical literature now describes numerous sex-based differences in immune responses, allergology is one of the disciplines in which this approach is still underdeveloped. The topic is complex and includes a spectrum of pathologies and manifestations that are very different from each other, while still few studies have investigated their sex and gender aspects. Data are fragmentary and sometimes discordant; in some cases there are only epidemiological observations, while in others the pathogenetic hypotheses are only speculative.

**Figure 1:**
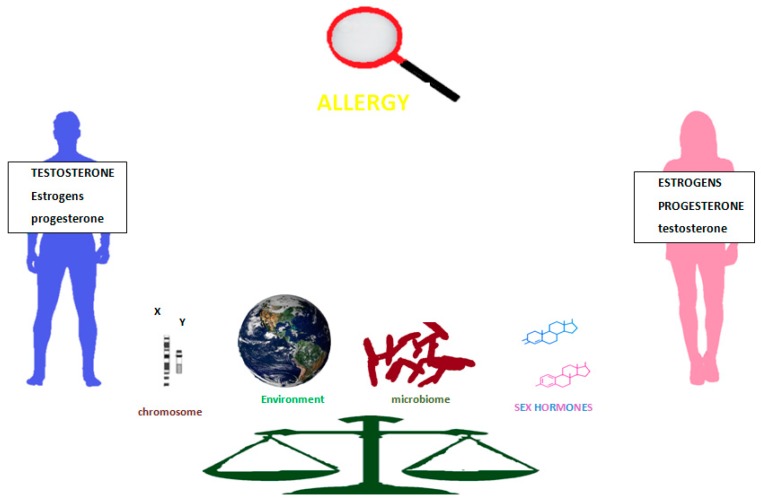
Sex, gender, and allergy: factors influencing susceptibility to allergic inflammation in females and males.

In this sex-gender vision, we summarize the knowledge of the pathogenetic mechanisms involved in allergies and sometimes shared by other immunologically mediated diseases. Then analyzing by disease or groups of diseases, the facts inferable from the literature, from time to time we will deal with epidemiological data, experimental findings, clinical observations and therapeutic results (Figure 1). Taking stock of the current knowledge, we aim to stimulate further research as the essential step for better development in allergology of personalized and patient-centred care.

## 2. Hormones, Chromosomes, Immunity, and Allergy

The complexity of most allergic diseases is based on a dynamic heterogeneous combination of hyperresponsiveness, dysregulated immune response, chronic inflammation, and tissue remodeling in affected organs [5].

Over several decades, clinical observations associated hormone status and allergic reactivity, but only recently, the mechanistic role of sex hormones in immune responses, has been acknowledged [6]. They contribute to the different sex based reactions in immune mediated pathologies. Males and females probably have different evolutionary needs, so the former have a more efficient humoral based response, whereas the latter show an immune response less efficient upon pathogen challenge [4]. Evidences indicate that progesterone, androgens, and glucocorticoids may have an immunosuppressive effect, whereas estrogens enhance mast cell reactivity, delayed type IV allergic reactions, humoral responses, and autoimmunity [7,8]. The male (testosterone) and female sex hormones (estrogen and progesterone) binding to the receptors expressed on the surface of a moltitude of cells types activate signaling cascade or directly induce gene expression [9]. Estrogen receptors α and β and G-protein-coupled receptors operate the estrogen effects, while progesterone works through two isoforms (A and B) of its receptor, and the androgen receptor NR3C4 mediates the effects of testosterone. These receptors may exist in different isoforms and have different downstream activity. Research highlighted the role of ERα in Th1, Th2, Th17, and Treg cells. In mice its interaction with transcription factors such as Foxp3 and GATA3 increases Treg and Th2 cells. Differentiation and function of Th17 is suppressed by the direct binding of Erα to the promoter region of retineic-acid-receptor (RAR)-related orphan receptor gamma (RORγt), one of the master regulators of these lymphocytes subset. Estradiol, probably through ERα inhibits Th1 and Th17 cells infiltration in an experimental autoimmune encephalomielitis model [10].

**Figure 2:**
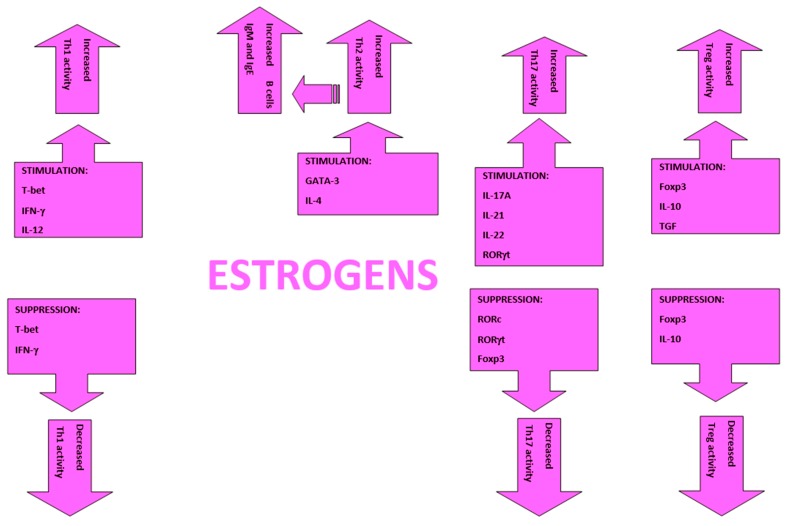
Show the effects of estrogens on the activities of Th1, Th2, Th17, and Treg cells [11]. Different effects may depend on hormone levels; for example, pregnancy or estrous levels.

Female sex hormon effects on T cells is complex and depends on concentrations, tissue and pathologic contest (Figure 2). Estradiol pregnancy levels for example inhibit Th1 development shifting towards Th2 profile the Th1/Th2 balance and reduce IL-12 and IFN-γ production. Furthermore increase on Th2 the GATA3 binding protein expression and IL-4 [11]. Estrogen receptors α and β are expressed in CD4+ T lymphocytes, and their signaling enhances Th1 production of IFN-γ while show variable effects on Th2 production of IL-4 and Th17 production of IL-17A. Estrogen and progesterone increase Th17 cells expression of IL-23R and IL-17A production, as well as increase airway inflammation mediated by IL-17A [12].

Estradiol leads to the proliferation and activity of Treg cells, inducing binding of the estradiol/ERα complex to estrogens response elements (EREs) on a human Foxp3 promoter, and thus enhancing the forkhead box P3 (Foxp3) expression. In-vitro induces the switch of CD4+CD25- lymphocytes in CD4+CD25+ with enhanced IL-10 and Foxp3 expression in a mouse model [11].

**Figure 3:**
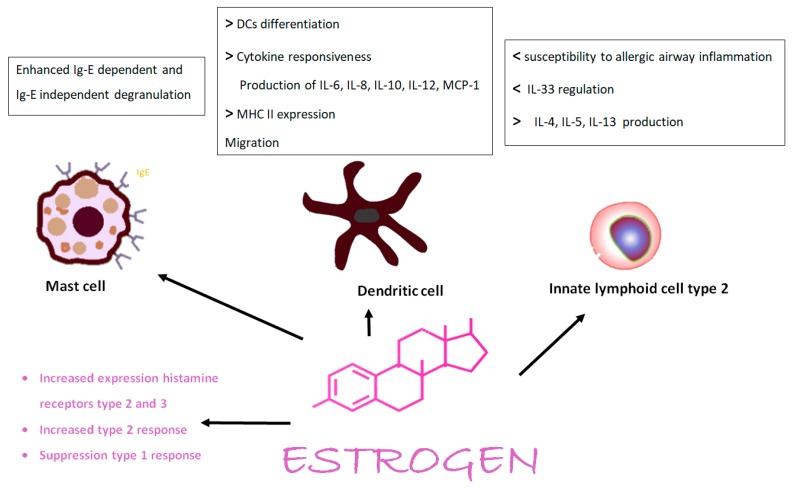
Estrogen’s influences on immunity.

Several observations such as the different prevalence and/or severity of several diseases in females and males endorse the general concept that testosterone inhibit immune function and estrogen and progesterone tend to enhance it. Estrogen impair negative selection of high affinity autoreactive B lymphocytes, modulating B cell function and leading to a Th2 response, promote lymphocyte B servival, maturation, class switching, and production of antibodies [13]. They also enhance the expression of CCR5, a homing molecule inducing T cell homing [14]. On the other hand, testosterone increase Th1 response and activate CD8+ cells and down regulates natural-killers responses and tumor necrosis factor [15]. This would result in an increased frequency of inflammatory and autoimmune diseases such as rheumatoid arthritis, systemic lupus erythematosus, and multiple sclerosis and reduced parasitic infections in females. These are some of the visible influences of sex disparities on outcomes and clinical manifestations of immune mediated diseases [16]. Females in general mount a vigorous immune response to vaccination, infections, and some malignancies [17]. It is now aknowledged that immune cells development and function are under sex chromosome-encoded genes or sex hormones regulation. A strong immune activation is observed in females through the action of estrogen and prolactine on T, B, and dendritic cells whereas testosterone stimulates myelopoiesis and favors the development of immunosuppressive neutrophils. The significant role of female hormones is also supported by the remission of systemic lupus erythematosus and rheumatoid arthritis during pregnancy. The sex hormones circadian rhythms and fluctuations throughout one’s entire life makes it very difficult to set up studies and interpret results to really understand their role in immunity and immune mediated diseases, and the elderly should not be overlooked since the inevitabile decline of sex hormones results in the many age related pathologies, including allergies [18]. In women a maintained elevated immune response and accumulation of antibodies over a period of time could induce low-grade inflammation, predisposing to sex bias inflammatory pathologies. Some prenatal events can contribute to sex specific differences in immune responses, and thus in higher susceptibility to immune diseases and infections in childhood and throughout one’s entire life [17]. Furthermore, variable responses in females and males result from the interaction of environmental factors such as infections and smoking and sex hormones. It is becoming evident that the sex hormones have an impact on microbioma and consequently on immune responses [15]. On the other hand, gut microbiota can modify the systemic levels of testosterone via 17β reduction of androgen but still lack adequate information on the mechanism by which sex hormones derived by microbiome impact on the host immune response [19,20]. Immune cell populations vary between the sexes: females from birth have a higher proportion of CD4+ T lymphocytes than male, CD4+ and CD8+ T cells decline with age in both sexes though compared to men, and aged women show lower memory Tregs and NK cells. These observations may at least in part justify the sex biased immune response and cytokine production. T cell activation and Th1/Th2 balance is regulated by both testosterone and estrogen. The effect of estrogen (Figure 3) appears to be dose dependent, with low dose inducing Th1. Estrogens may also promote in CD4+ T cells the expression of IL-21 and increase B cell proliferation and induction of Th17 [4]. They increase IgE production in mouse spleens [21] and in humans several B cell subsets are more numerous in females than in males. Progesterone enhances the proportion of B regulatory cells that produce IL-10, which may prevent in the airways the allergic Ig-E mediated response of mast cells. Testosterone directly prevents B cell maturation and inhibit antibody production. Major histocompatibility complex (MHC) molecules generate immune response presenting antigens from pathogens. Estrogens increase MHC II expression on dendritic cells (DCs) while testosterone decreases their expression. Considering the role of DCs in generating the immune response and T cell differentiation, there is evidence of a sex specific driven response. Estrogens influence their capacity of producing IL-6 and IL-12 and migration. Progesterone could inhibit DC activation and their ability to activate CD4+ T cell proliferation in a dose dependent manner. Testosterone appears to have similar activity, including the inhibition of DCs to produce proinflammatory cytokines. IgG4 were found at a higher concentration in healthy adult males, and this molecule has a blocking effect against mast cells stimulation, probably binding to allergen and avoiding its binding to IgE on mast cells [22].

In allergic reactions, mast cells have a leading role and have been shown in a mouse experimental model to have sexually dimorphic responses [23]. In other studies on the expression profile of histamine receptors was found a gender difference with histamine receptor 1 equally expressed in males and females, while histamine receptors 2 and 3 are highly expressed in females [23]. Male sex hormones negatively influence another key regulator of type 2 inflammation, the group 2 innate lymphoid cells. Males show a reduced number of these cells with lower susceptibility to allergic airway inflammation after environmental allergen stimulation and a reduced interleukin-33 mediated lung inflammation [23,24]. Toll like receptors (TLRs) are differently expressed between sexes and can influence TLR dependent response strength: TLR2 and TLR4 have a higher expression in males, TLR3, TLR7, and TLR9 in females [25,26]. During infections in male mice, macrophages induce a stronger TLR2 and TLR4 dependent Th1 response, whereas estrogen modulates endosomal TLRs and TLR8 expression. TLR2 and 4 bind bacterial cell proteins while TLR 3, 7, and 9 recognize viral DNA or RNA [15,27]. Compared to women, mononuclear cells from men produce higher levels of interleukin 10 in response to TLR9 ligands and lower level of type I interferons responding to TLR7 ligands. Eicosanoids the potent bioactive lipid mediators mostly produced by immune cells through the pathways of the cyclooxygenase, lipoxygenase and cytochrome p450 regulate acute and chronic inflammation and play a crucial role in normal and aberrant immune responses. Belonging to this family of molecules are prostaglandins, leukotrienes, hydroxyeicosatetraenoic acids, and dihydroxyeicosanotreic acids, which spread the inflammation and anti-inflammatory lipoxins, epoxyeicosatrienoic acids, and E-series resolvins. Eicosanoid formation is induced through stimuli on receptors strongly modulated by sex. The pro-inflammatory eicosanoids production is sex biased, indeed testosterone regulating the subcellular localization of the 5-lipoxygenase, a key enzime, drive the formation of leukotriene [28]. Several studies suggest that androgens suppress leukotriene levels, and female sex is associated with higher biosynthesis of these molecules. Hence, the suggestion of a possible gender tailored treatment of leukotriene related disorders such as asthma and allergic rhinitis [29]. Montelukast a cysteinyl leukotriene receptor 1 antagonist reduced days with worse asthma symptoms in girls reaching puberty respect to peers boys in a clinical trial [29], so it appears to be more beneficial in women than in men [30]. In men rather than in women, preponderate prostanoids and their biosynthetic enzymes, however, are also relevant to the hormonal status in relation with the age of studied people as well as the type of organ and tissue. Summarizing that while androgens possess overall anti-inflammatory activities, being reported to exert multiple immunosuppressive action and to impair autoimmunity, the anti-inflammatory activity of estrogens depends on their timing and concentration, the immune stimulus, target organ, cell type involved, the estrogen receptor subtype expression, and intracellular metabolism of estrogens [29].

Sex bias in immunopathology may reside in gene diversity or dosage. Several genes playing a critical role in the regulation of immune response and among them that encoding for FoxP3, CD40L, TLR7, TLR8, GATA1, and IL-1R associated kinase 1, IL-13 chain, Il-2R chain, Il-3R chain, are located on chromosome X and while men have only one X, women carry two copies of this chromosome of which one is randomly transcriptionally inactivated [31]. Paternal or maternal X inactivation in different cell types in females and the presence on this chromosome of genes associated with immune regulation could reasonably suggest more than a simple involvement in sex bias immunity [32]. Hormonal and immune dysfunctions are observed in inherited disorders with X chromosome involvement such as females Turner (X0) and males Klinefelter (XXY) sindrome. Furthermore, the X chromosome contains 10% of microRNA in the human genome as compared to 2 on the Y chromosome and the X linked microRNA leads to much higher immune responses in female participating in sex differentiated immunity [33]. Authophagy may be another of the dysregulated basic cellular processes [34] involved in the pathogenesis of allergies. Estradiol regulates autophagy in several cell types and may activate the phosphoinositide 3-kinase (PI3K)/Akt pathway [35,36]. It is an homeostatic process with an emerging leading role in causing downstream changes triggered by allergens and environmental polluttants in allergic asthma [37]. Through IgE production and antigen presentation, B cells, also under the influeces of sex hormones [4], play important roles in the inflammatory process of allergic asthma and it is suggested that through multiple mechanisms B lymphocytes autophagy aggravates experimental asthma [38].

The kinase mTOR intervene in T helper growth, proliferation, and metabolism. Th1, Th2, and Th17 differentiation depends on mTOR activity while its inhibition favors Treg differentiation [39,40]. mTORc1 activation is required for differentiation towards all effectors lineages including Th2, since its inhibition through RAPTOR deletion powerfully interrupt the process and induces Th17 differentiation [41,42]. Deletion of Ras homolog enriched in brain (RHEB), a positive regulator of mTORc1, arrests Th1/Th17 but not Th2 differentiation [43]. In response to cytokine stimulation activation of mTORc1 induces Th1 and Th17 differentiation through activation of the signal transducer and activator of transcription (STAT) and downregulation of early growth response 1 a negative regulator of growth factor independent 1 (Gfi1) expression. Conversely, Th2 differentiation is inhibited by mTORc2 through inhibition of the suppressor of cytokine signaling-5 (SOCS5) that blocks in cascade IL-4-dependent Signal Trasducer and Activator of Transcription-6 (STAT6) signaling. The downstream target of mTORc2 serine/threonine-protein kinase (SGK1) blocks Th1 cell lineage commitment and and promotes Th2 development. Furthermore deletion of another target of mTORc2 the GTPase RhoA reduces glycolisis and IL-4 secretion and Th1, Th2 and Th17 growth and fuction is highly glycolytic [39,44]. To enhance Tregs generation in normally activated conditions, the simultaneous inhibition of mTORC1 and mTORc2 [41,45] is required.

With reference to B cells, the mTORc pathway is certainly essential for many aspects of the B lymphocyte development and function, but much of details of these regulatory circuitry are still to be clarified [46].

## 3. Asthma and Rhinitis

Asthma is a significant public health issue affecting over 300 millions people worldwide. Increased airway reactivity, inflammation, and mucus production drive coughing, wheezing, shortness of breath, and/or chest tightness, resulting in a clinically heterogeneous disease that ranges from mild to intermittent to severe asthma [38]. It is the result of environmental exposure with genetic and hormonal factors. The complex interaction of endogenous and exogenous sex hormones with obesity and external exposure modulate gene transcription and acts on airway inflammation and reactivity. Females and males are differently affected [47]. More prevalently and severely in pubescent males after puberty, there occurs a gender switch. In men aged 45 or more, increases in the risk of asthma severity and in the elderly differences related to gender diminish [48]. The MeDALL study showed an higher prevalence of asthma, rhinitis, and respiratory multimorbidity in boys before puberty and a sex balanced estimated prevalence after puberty [49]. For asthma as a single entity, the female prevalence that arises after puberty persists in adulthood. Less clear are sex specific prevalences before and after puberty [50,51]. Lungs in females are lighter and smaller than in males and have a lower total number of alveoli with higher forced expiratory flow rates [52]. The menstrual cycle, pregnancy, and exogenous sex hormones supplementation, in females, affect asthma. Sexual differences in asthma also result from gene polimorphisms including sex specific asthma risk loci and thymic stromal lymphopoietin (TLSP) and estrogen receptor alpha (ESR1) gene polimorphisms [53]. Estrogens up regulate and androgens down regulate Th2 responses. Sex, asthma, and obesity interact and represent an interesting and growing field of research, appearing women to suffer for the effect of obesity on asthma severity while some obese women develop late onset asthma. Both men and women may be affected by asthma complicated by obesity or asthma as a consequence of obesity that prevails in women. A sex specific effect of obesity is observed in the pediatric population with obese children showing lower prevalence of bronchial hyperreactivity than non-obese children and an higher number of obese girls respect to boys. In girls but not in boys, a positive association was shown in longitudinal observational studies between body mass index and asthma like symptoms. It is supposed that in this asthma interaction in obese children, may play a role a difference in immune responses to environmental triggers, differences in lung development and symptoms perception. Also, obese adult females tend to show higher prevalence, incidence, and severity of asthma. The precise mechanism underlying sex specific association of asthma and obesity is still unclear, but recently was reported that human airway smooth muscle cells from obese subjects aged 20–40 had greater cell shortening and greater response to contractile agonists in females than in males [53]. Body fat percentage is lower in males than in females with the same body mass index, and they have higher percentage of visceral fat while the latter have more subcutaneous fat especially in the gluteal-femoral area. The gender difference in visceral fat distribution is controlled by an estradiol-autophagy axis [54]. Lower pulmonary function and higher risk of restrictive respiratory defect are associated only with central adiposity and not with general obesity and these observations may contribute to the sex differences in asthma risk and severity. Furthermore, adipose tissue represent a highly active endocrine organ whose molecule production profile change between the two sexes and differently modulates systemic inflammation. Adipokines play a leading role in connecting obesity and systemic inflammation: adiponectin and leptin are associated with obesity and are both lower in adult males in respect to females with the same body mass index. Fat distribution and testosterone level partially explain these differences. In animal models there is an association between high leptine levels and airway hyper-responsiveness. Lower levels of adiponectin are associated with asthma. A further connection between asthma and obesity appear to be insulin resistance as shown in adults females with polycystic ovarian syndrome in association with ovarian hyperandrogenism and obesity [55].

In obese adult females, neutrophilic inflammation is suggested to play a significant role: were found high neutrophil counts in peripheral blood samples and sputum from obese asthmatic women and not in men [53]. E-Lacerda et al. showed in a mouse model that the interaction between weight gain and sex alters the progression of allergic asthma: females develop airway remodeling at an earlier stage in respect to males [56]. In female most studies report lung eosinophil infiltrates, higher serum IgE production and type 2 cytokine lung production than in male [57], and this is linked to increased airway hyperresponsiveness and remodelling. Inflammation in asthma is mediated by type 2 cells, IL-17A, and IFN-gamma secreting cells. Increase of CD4+ Th2 cells, group 2 innate lymphoid cells, mast cells, basophils, eosinophils and other types of cells, interleukin (IL) -4, IL-5, IL-13 and IgE production, characterize type 2 airway inflammation [58,59]. Enhanced secretion of IL-17 from CD4+ Th17 cells, gamma/delta T lymphocytes, neutrophils and group 3 innate lymphoid cells in the airways characterize IL-17 airway inflammation that is associated with more severe phenotypes of asthma. CD4+ Th1 cells, cytotoxic killer T cells and natural killer cells mediate the IFN-gamma airway inflammation. Several mouse models of asthma were helpful to elucidate some of the mechanisms by which sex hormones regulate airway inflammation [60]. Th2 mediated airway inflammation is decreased by testosterone and increased by estrogens but the hormonal regulation of other pathways is still under investigation. Takeda et al. demonstrated a female predominance in the production of IL-10, IL-13, TGF-beta and PDGF [61]. Airway remodeling is of critical significance in developing severe asthma and is the result of immunologic and inflammatory mechanisms influenced also by sex. In a mouse model was documented an increase of cellular and humoral markers of inflammation in bronchoalveolar lavage and enhanced histological features of airway remodeling in females compared with males [23]. Young adults with allergic asthma show increased number of circulating mast cell progenitors related to female gender, high levels of fibroblast growth factor 21 (FGF-21) and reduced lung function [62].

Being implicated in immune function [36], the mTOR pathway have a role also in asthma: during asthma onset mTOR is activated and during remission suppressed. The inhibition of the mTOR pathway in asthmatic mice lowers asthmatic markers and refreshes the balances of Th1/Th2 and Th17/Treg cytokines [63].

Furthermore, Zhu et al. showed that mTOR inhibition suppress eosinophil differentiation in allergic airway inflammation [64], Shin and coll. that reduces eosinophil infiltration and Th2 immune response [65] and Fang et al. that IgE induced airway smooth muscle cell (ASMC) remodeling was significantly reduced by inhibition of mTOR [66] while Rakhmanova et al. suggested as a therapeutic strategy for mast cell-related diseases the hyperactivation of mTORC1 [67]. The greater role of airway inflammation and remodeling driving severe asthma in women and the regulatory action of estradiol on activation of PI3K/Akt signaling pathway [35], suggests a possible gender specific function of mTOR inhibition.

## 4. Food Allergy

Food allergy is characterized by different immunologic pathways and natural history and involve complex gene-environment and social interactions. Sex, age, psychological and socio-economic factors influence the developing and the manifestations of these disorders. IgE-mediated such as oral allergic syndrome and digestive anaphylaxis and non-IgE-mediated such as eosinophilic disorders and food protein induced enterocolitis syndrome food allergies are described and represent an increasing public health concern [68,69,70,71]. It is recognized the association of food allergy with some congenital immunodeficiency syndromes. Examples are a variant of immune dysregulation polyendocrinopathy X-linked syndrome due to a mutation in the forkhead box P3 gene (FOXP3) in which most patients are characterized by the clinical triad of intractable diarrhoea, insulin-dependent diabetes and eczematous dermatitis but some of them, show severe food allergy and hyper-IgE autosomal dominant syndrome with dedicator of cytokinesis 8 (DOCK8) gene mutation. Natural history of food allergy may change. It is known that peanut and tree nut, or allergy to seafood and fish diagnosed in the first years of life are mainly persistent. Cow’s or hen’s eggs Ig-E mediated allergy diagnosed in children frequently recover in the first year of life but also in subsequent years. The risk of persistence could be associated to high specific IgE levels and/or an allergic respiratory comorbidity. Age related sex differences are reported in food allergies similarly to atopic disease, hayfever and asthma confirming the relevance of the impact of puberty and the influence of sex hormones [72]. Peters and coll. showed in the pediatric healthnuts cohorts that male sex was strongly associated with the two multiple food allergy phenotypes(predominantly peanut and predominantly egg), but not single egg allergy [73]. The Mirabel study showed an age dependent sex distribution with a male/female sex ratio of 1.50 in children in contrast to early adults where the ratio was 0.67 [74]. A questionnaire based survey assessed that food induced complaints are more frequent in females 24% than in males 14% [22]. More recently data from an electronic health records allergy module showed a significant predominance of females with adverse food reactions (4.2%) respect to males (2.9%) [75]. More females having the severe phenotypes of peanuts allergy seem to be more frequent in females as a consequence of a possible gender effect. Offending foods seem to be characterized by sex-gender variations, these are specifically described with fruits and berries that more frequently trigger adverse food reaction in women (44%) than in men (24%), whereas paenuts appear to be more frequently responsible as trigger of allergic reaction in males (43%) than in women (27%). A positive skin test for food allergens is more frequent in females (27.5%) than in males (22.5%), more evident for peanuts (females 20.4%, males 15.2%) and pollen associated food like celery (females 17.2%, males 12.1%) [22]. These data suggest that in the diagnostic phase it could be necessary to adapt the progressive dosage of provocation tests considering the female gender and the allergic multi-morbidity [74]. The most effective management of food allergy remains to be strict avoidance of the identified allergen and also in this context a gender issue should be considered: more females (4.8% and 7.4%) adhere to a strict allergen free diet than males (2.3% and 4.1%) in two different studies [22]. In food allergy as in other allergic conditions several factors may influence the female prevalence: awareness of risk, health knowledge, cultural differences, sex hormones, self-reporting, psychologic sensitivity, processing information, history interview with physician, usage of specific medication (e.g., anti-acid medications), efficiency of medication.

## 5. Atopic Dermatitis

Pruritus, skin barrier impairment, and T helper 2 (Th2) shifted abnormal immunity characterize atopic dermatitis (AD) a chronic inflammatory skin disease [76]. In childhood is reported a slightly higher prevalence in boys (8.7%) than in girls (5.6%) [11]. After puberty females predominate on males: respectively 8.1% and 5.7% in a study in Japan and 8.01% and 6.04% in a study in Europe and USA [24]. It was suggested that sex hormones may contribute to the higher incidence of AD-like dermatitis in female rats KFRS4/Kyo [77]. In intrinsic AD, in all generations exist a female predominance with increased Th1 activity and high incidence of nickel allergy and without filaggrin mutation or enhanced serum IgE levels. Further observation is that AD deteriorate when both estrogen and progesterone are secreted in the luteal phase, before menstruation [24]. Is reported a deterioration of AD during pregnancy that may reflect the effects on skin barrier and Th2 activity of extremely high levels of estradiol and progesterone.

In adult females, skin hydration is slightly higher than in males and men have a significantly higher basal transepidermal water loss respect to women. Androgen and progesterone enhance skin barrier impairment while estrogens strongly suppress. Estradiol and or progesterone directly or indirectly induce the secretion of IL-4, IL-13, IL-31, IL-33, Th2-related cytokines [24] generate an itch sensation binding the corresponding receptors on C-type sensory neurons. Estrogens also promote release of histamine by mast cells. Furthermore, women appear to be more responsive than men to pruritogens.

## 6. Urticaria

Chronic urticaria (CU) is a common dermatologic condition characterized by pruritus, redness swelling, and wheals with important repercussion on the quality of life [78,79,80]. Limited data are available on a sex-gender perspective of CU. With an average female to male ratio of nearly 2-4/1, CU is included among the skin disorders that have a significative predilection for females [81]. To date, no hypothesis has been formulated on the pathogenetic mechanism that determines the female prevalence of this disease. Most of the immunological features characterizing the female and described so far, certainly play a role even in urticaria.

Although being more frequent, we have no data on the gender distribution of acute urticaria [23].

## 7. Anaphylaxis

Anaphylaxis is a severe and virtually fatal generalized or systemic hypersensitivity reaction with usually at least two systems involved between respiratory, cardiocirculatory, gastrointestinal and skin and characteristically rapid in onset. The rapid release of mediators by mastcells as part of an IgE mediated type I hypersensitivity represent the main trigger of anaphylaxis but several others not strictly related to allergy were identified and idiopathic anaphylaxis is described [82]. Hormonal and sex dependent factors may have a leading role in anaphylaxis but to date the female predisposition towards immunological immediate reactions still miss confirming data. In fact only some experimental research has been conducted to address the gender issue in anaphylaxis: in female mice this reaction is more severe than in male, estrogen upregulate endothelial nitric oxide synthase (eNOS) and mast cell degranulation increasing vascular permeability and systemic consequences [83,84]. In humans mast cells are affected in vitro by progesterone and estrogen, but their in vivo effects are still undetermined while a possible sex hormones influece may indirectly be suggested by the rare breastfeeding and catamenial anaphylaxis [85,86]. In women is reported an increased risk of anaphylaxis than in men but the validity of these studies have major biases such as definition of anaphylaxis, cohort composition, self-reported events, the lacking of homogeneous criteria and uniform coding system [82]. Anaphylaxis by all causes has been reported with higher frequency in adult females than in males and this predominance appear after puberty. However, there is no sex difference in rates of fatal anaphylaxis [87].

## 8. Drug Allergy

Speaking of adverse drug reactions (ADRs), we refer to all types of reactions related to drugs including side effects. Only a small proportion of adverse drug reactions have an immune-mediated basis induced by adaptive or innate mechanisms [88]. Self-reported drug allergies are mostly not true allergy and rarely investigation identify a pathogenetic immune mediated mechanism. ADRs are more frequently reported in women or they occur more severely in women [89]. In adult females are more frequently self-reported drug allergies and immunologically mediated ones. In children, this female predominance is not observed. Female propensity to drug allergy may be related to several possible mechanisms: different utilization of health care, exposition to medications, genetic factors linked to chromosome X, epigenetic changes, hormonal influences on immune cells [90]. Studies on large populations showed higher allergy prevalence rates for all classes of drugs and penicillins, followed by sulfonamides and non-steroidal anti-inflammatory drugs are the main responsible [89]. A female predominance is reported in cases of reaction to anesthetic and anti-epileptic drug, radiocontrast media and perioperative anaphylaxis [91,92,93], however large scale studies are needed. Not all studies agree on the female predominance of radiocontrast reaction and women seem more vulnerable to more severe reactions [94]. A higher frequency of anaphylaxis to medication in women and in the elderly was attributed to more exposure to medication, it is possible instead that the age is responsible for the increased vulnerability to develop immune response to drugs. Drug induced anaphylaxis is most frequent in females, but in children there is no clear sex predominance [95]. Also vaccine induced anaphylaxis show a female predominance after puberty and a male predominance in pre-puberty [96,97].

## 9. Vaccines

Females have been shown to have an immune privilege in response to vaccinations. Indeed, the response to vaccination differs between sexes and appear in general stronger in women. The counterpart of this evidence, unfortunately, is that women experience more often and worse side effects of vaccination than males. The hypersensitivity reaction may consist of urticaria, pruritus without rash, dyspnea, wheezing, angioedema, flushing, rhinitis, syncope, dizziness, hypotension, nausea, diarrhea, and/or vomiting up to anaphylaxis. Causal relationship exist between anaphylaxis and vaccines, though adverse events following vaccination are rare and anaphylaxis very rare with a rate of occurrence of about one for million for most vaccines [98]. The higher frequency of hypersensitivity reported in women may depend both on a more frequent experience of adverse events or to a better predisposition to report the events respect to men [99]. Literature data while suggesting a predominance of women, they have some limitation due to passive surveillance and rarity of events. Large-scale population studies will clarify differences rates by sex-gender and related biological basis.

## 10. Conclusions

In this review we have summarized and discussed sex-gender aspects that characterize allergic diseases to give an updated report on this topic. We tried to look through pathogenetic mechanisms, epidemiological, and clinical data to highlight the most up-to-date knowledge. The complexity of allergic inflammation in its several forms [100] supports the need for recognizing sex-gender associated differences to better understand the pathobiology of the allergic diseases. The ultimate goal is to define and understand these differences or their absence and improve prevention, diagnosis, care and treatment of both women and men.

Recently the European Academy of Allergy and Clinical Immunology (EAACI) and the American Academy of Allergy, Asthma and Immunology, (AAAAI) evaluated the potential to utilize a precision medicine approach to anaphylaxis, food and drug allergy and to airway and skin allergic diseases, recognizing that progress has been made, but that much remains to be done [101].

Labella et al. confirmed that classification based on phenotypes, endotypes, and biomarkers of anaphylaxis allows for a better understanding of its mediators, mechanisms, and presentation [102].

In this view, it is indispensable to systematically investigate sex disparities, possibly in different age groups, allergic diseases incidence, and outcomes. When they are identified, it is necessary to clarify their biological basis and understand if improved outcomes could be achieved with sex-specific treatment modifications [5].

It is necessary to promote sex–gender awareness and prioritize sex–gender research, evaluating sex–gender differences and similarities over the human lifespan, including pre-natal life. Therefore, there is a need for new and reliable study design and modeling that integrates and considers both the biological differences and psycho-social, cultural, and economic factors that can affect men and women differently. This means a multi-dimensional patient assessement keeping in mind the unity of psycho-social (gender) and biological (sex) aspects [103,104].

Most of the immunological aspects driven by sex hormones are the fundamentals for the development of allergy, chronic inflammation, and autoimmune disorders that show a striking female sex bias [105]. Similarly to allergies, autoimmune diseases define proper phenotypes across puberty, pregnancy, menopause, and more in general in aging, linking the complexities of immunity and sex hormones. These became essential stratification criteria to better identify individualized patient treatments towards the so-called personalised medicine [106].

The heterogeneous nature of allergic-inflammation generates several doubts for the development of treatments targeting specific pathologic mechanisms that may only be observed in some patients [107,108].

We have the urgency to establish criteria that identify different categories of patients who need different therapeutic approaches, focused on specific targets.

Certainly one of the criteria currently known but still neglected is that of sex-gender, which represents a basic perspective to realize a tailored medical approach [106].

To effectively apply a preventing, predictive, and personalized medicine plan, Qian et al. [109] suggest an individualized patient profiling. Similarly, Golubnitschaja et al. [110] in a case study on Flammer Syndrome trace in detail how to conduct an individualized patient profile analysis, and Baban et al. [111] give an example method to unraveling common denominators between overlapping conditions [106].

Recently, Roberts et al. [112] described developments in the field of clinical allergy in 2018 and while they look forward to further advances for 2020, we associate ourselves with this wish, but feel the need to point out that an appropriate gender perspective is still lacking in allergology research. We can only add and encourage development in this direction.
